# Supplementary material for: Retrotransposons as pathogenicity factors of the plant pathogenic fungus Botrytis cinerea
Source: Genome Biol. 2021 Aug 16;22:225. doi: 10.1186/s13059-021-02446-4 (PMC8365987; doi:10.1186/s13059-021-02446-4)
Supplement: Supplementary file 1 — Additional file 1:. Figures S1-S15. [file 13059_2021_2446_MOESM1_ESM.pdf]

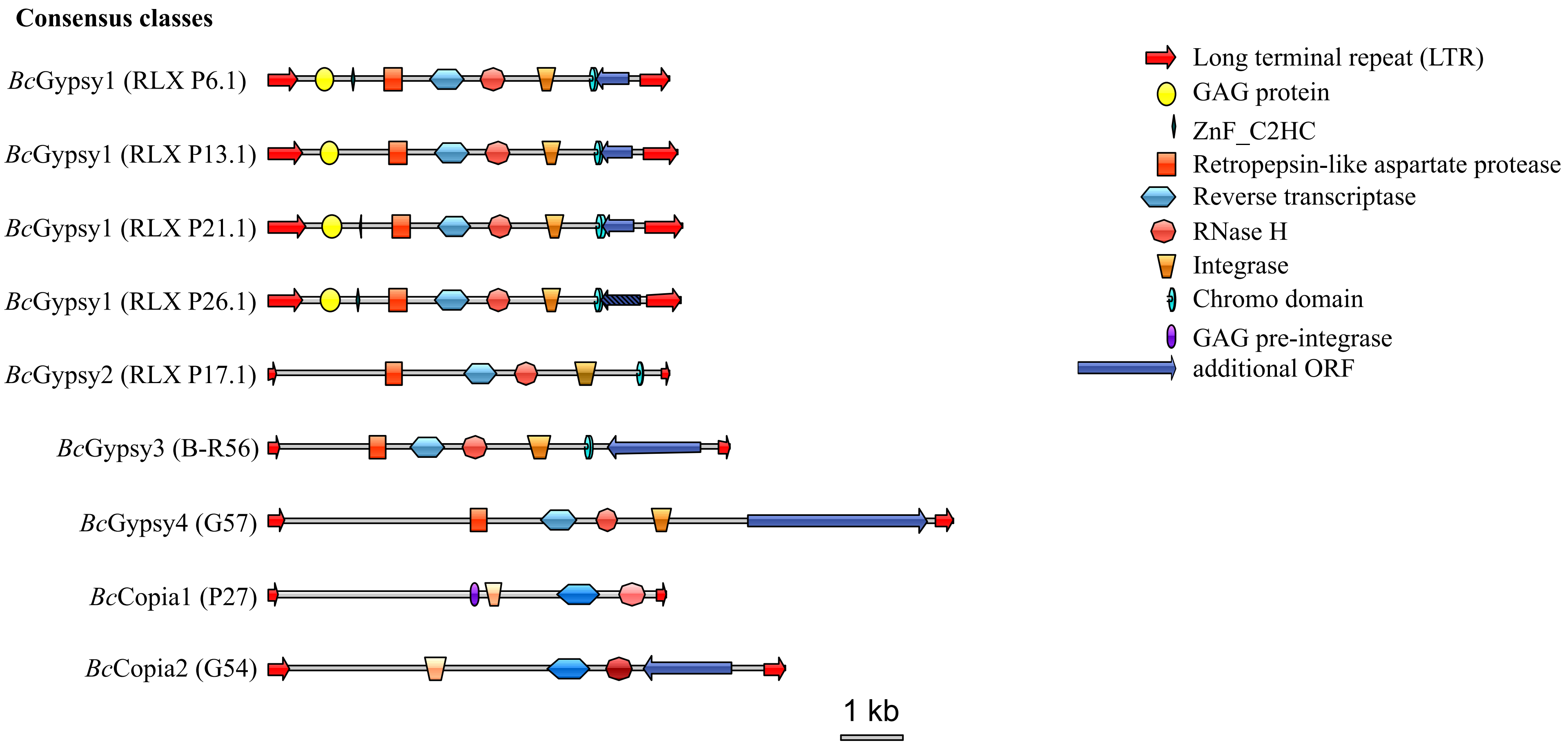

Figure S1: Conserved domains of LTR retrotransposons in B05.10 representing the nine consensus classes and the six subfamilies.

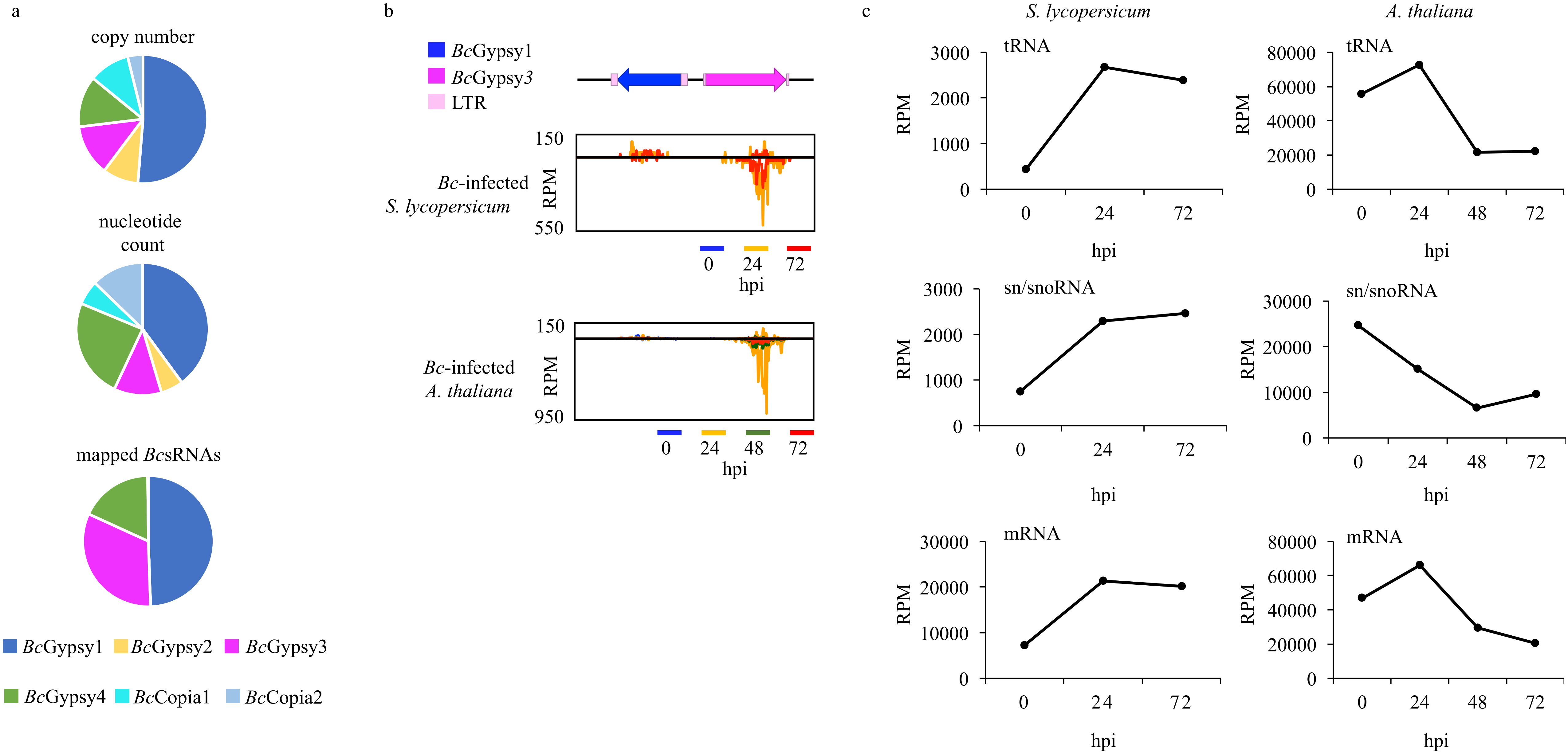

Figure S2: a) Copy number, nucleotide count, and mapped *Bc*sRNAs of the six LTR retrotransposon subfamilies *Bc*Gypsy1-4, *Bc*Copia1-2. b) *Bc*sRNA maps at the *BcGypsy1*/*BcGypsy3* locus at different time points of *A. thaliana* or *S. lycopersicum* host infection. Bars above line represents sense and below line antisense reads. c) *Bc*sRNA abundance in reads per million total *Bc*sRNAs mapping to tRNA, sn/snoRNA or mRNA gene loci at different time points of *S. lycopersicum* or *A. thaliana* host infection, with 0 hpi implies *B. cinerea* spore inoculation and direct sample harvest. For b) and c), raw data are available at NCBI GEO: GSE45323, GSE45321.

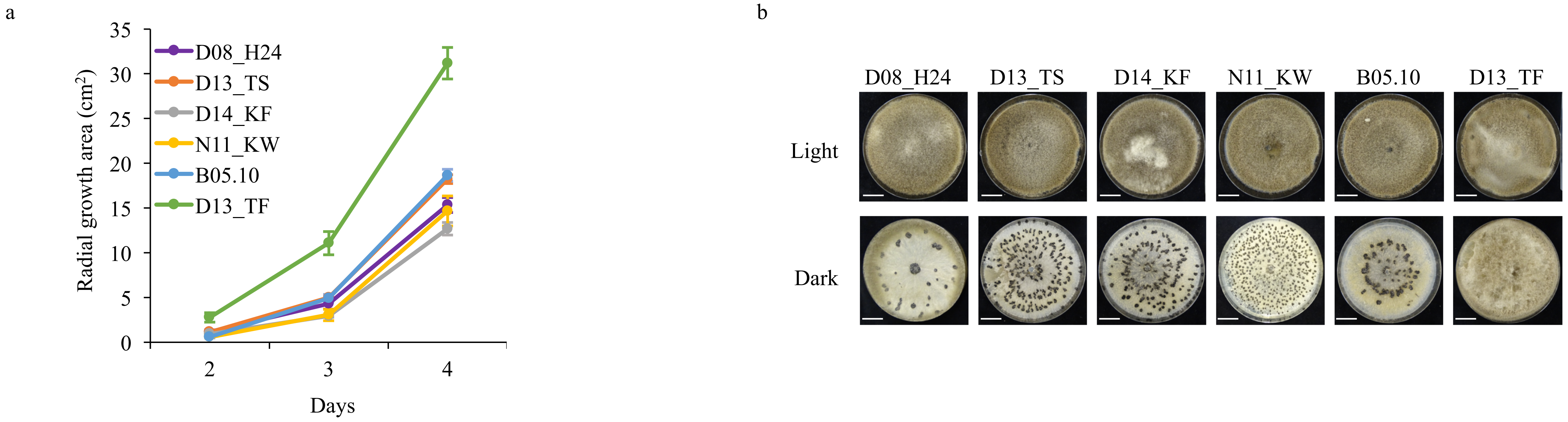

Figure S3: a) Radial growth speed and b) sclerotia formation of the six *B. cinerea* strains. Scale bar in b) represents 2 cm. For a), error bars represent standard deviation from three biological replicates.

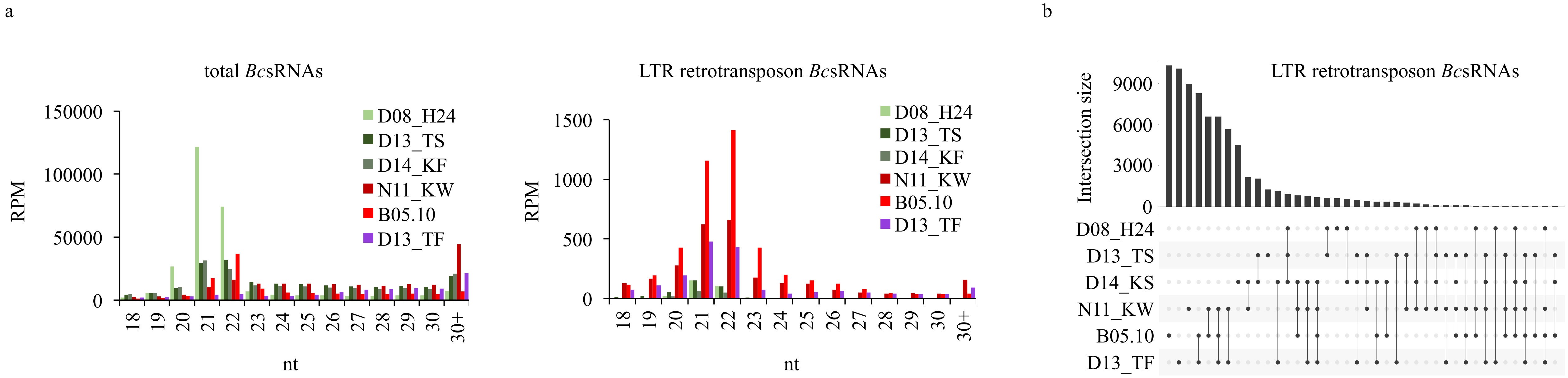

Figure S4: a) Unique reads size profiles of total *Bc*sRNAs and LTR retrotransposon *Bc*sRNAs in the six *B. cinerea* strains. b) UpSet plot displaying the intersection size of *Bc*sRNAs among the six B. *cinerea* strains.

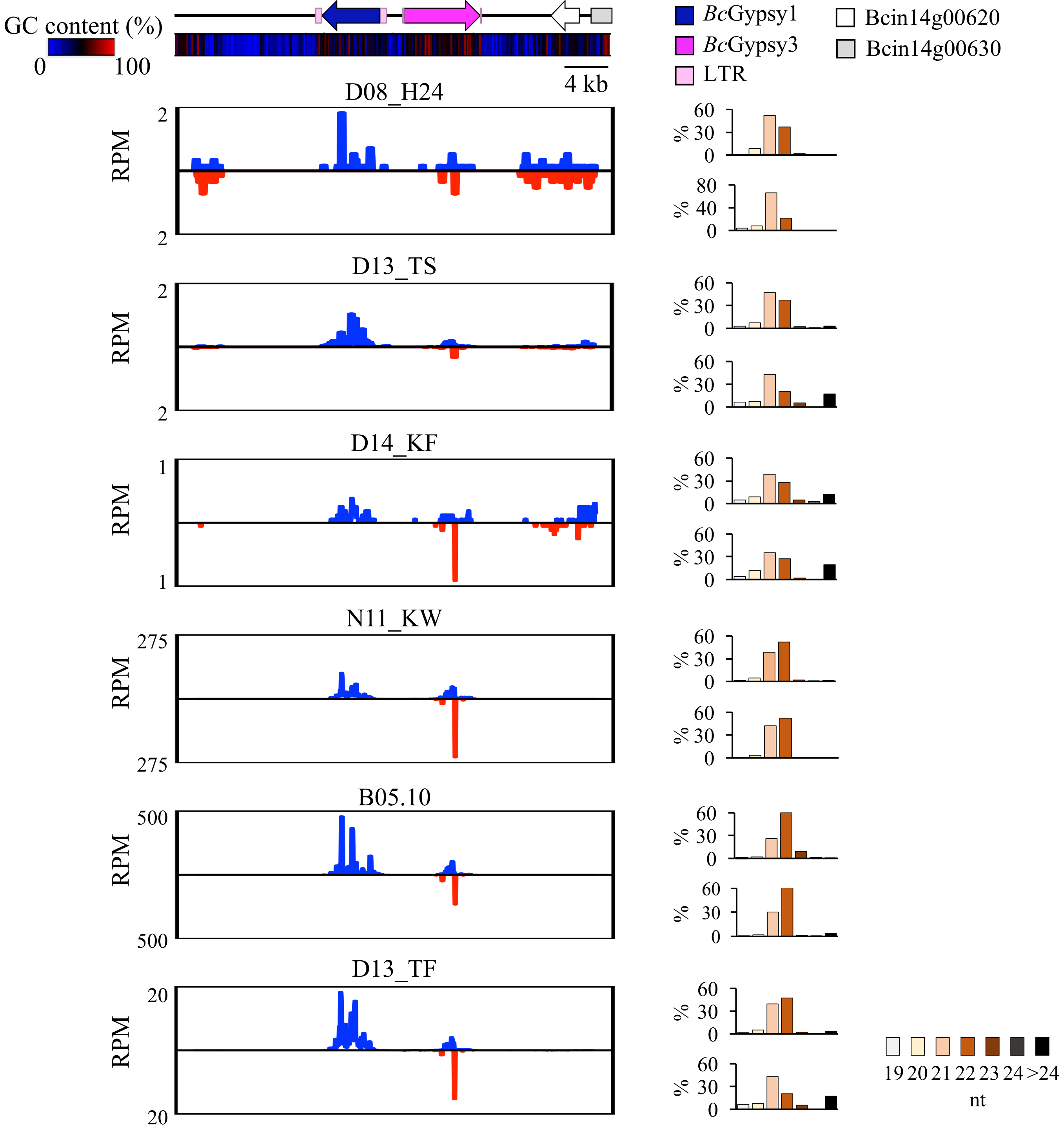

Figure S5: *Bc*sRNAs of the six *B. cinerea* strains mapping to a *Bc*Gypsy1/*Bc*Gypsy3 locus in B05.10 exemplifying reads aligned in sense and antisense are represented in blue and red, respectively, and the bar plots represent the percentage of reads with a specific nucleotide length. Different RPM scales are shown; 275 RPM for N11_KW, 500 RPM for B05.10, 20 RPM D13_TF, 1 RPM for D14_KF and 2 RPM for D13_TS and D08_H24.

a

	B05.10	D08_H24	D14_KF
*Bc*Gypsy1	357,565 bp<br>0.84 %	92,081 bp<br>0.22 %	164,229 bp<br>0.38 %
*Bc*Gypsy2	49,141 bp<br>0.12 %	21,556 bp<br>0.05 %	64,674 bp<br>0.15 %
*Bc*Gypsy3	103,916 bp<br>0.24 %	7,952 bp<br>0.02 %	37,056 bp<br>0.09 %
*Bc*Gypsy4	217,377 bp<br>0.51 %	45,201 bp<br>0.11 %	77,969 bp<br>0.18 %
*Bc*Copia1	53,275 bp<br>0.13 %	31,145 bp<br>0.07 %	7,152 bp<br>0.02 %
*Bc*Copia2	114,550 bp<br>0.27 %	22,194 bp<br>0.05 %	70,416 bp<br>0.16 %
**Total coverage**	**895,824 bp**<br>**2.10 %**	**220,129 bp**<br>**0.52 %**	**421,496 bp**<br>**0.99 %**

Figure S6: a) Genome coverage of LTR retrotransposon subfamilies in the strains B05.10, D08_H24 and D14_KF. b) Copy length distribution of LTR retrotransposons in the strains B05.10, D08_H24 and D14_KF.

b

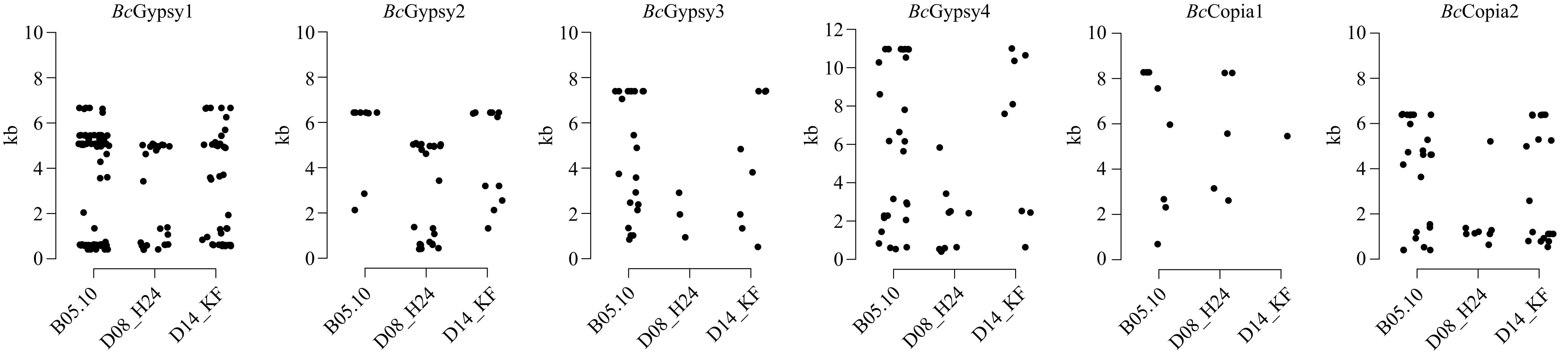

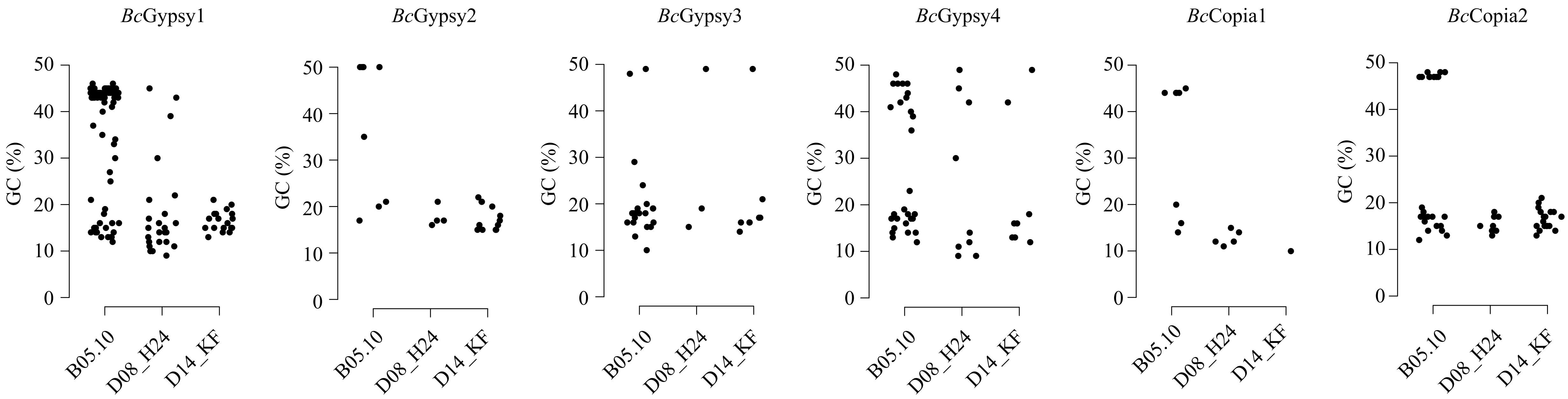

Figure S7: Analysis of GC content (%) of truncated LTR retrotransposons identified by Blastn search.

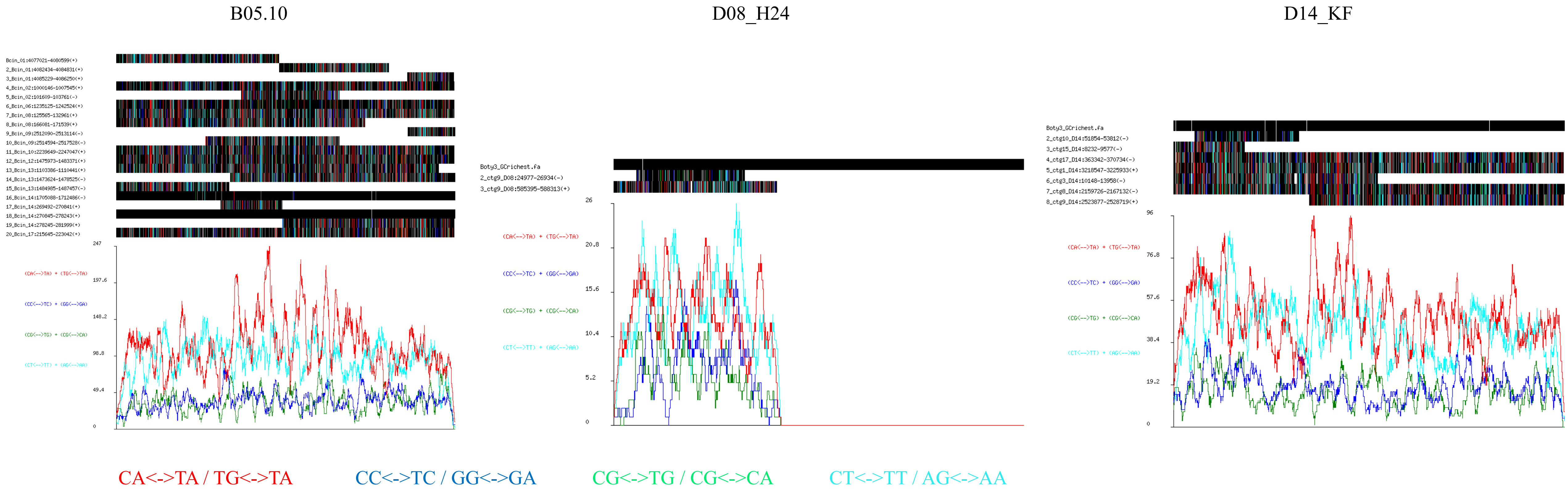

Figure S8: RIPCAL analysis of the of the *Bc*Gypsy3 subfamily in the strains B05.10, D08_H24 and D14_KF. Similar results were found for all six LTR retrotransposon families, *Bc*Gypsy1-4, *Bc*Copia1, *Bc*Copia2. Each line represents one *Bc*Gypsy3 copy. The GC-richest *Bc*Gypsy3 copy of B05.10 was used as reference for alignment with *Bc*Gypsy3 copies in D08_H24 and D14_KF.

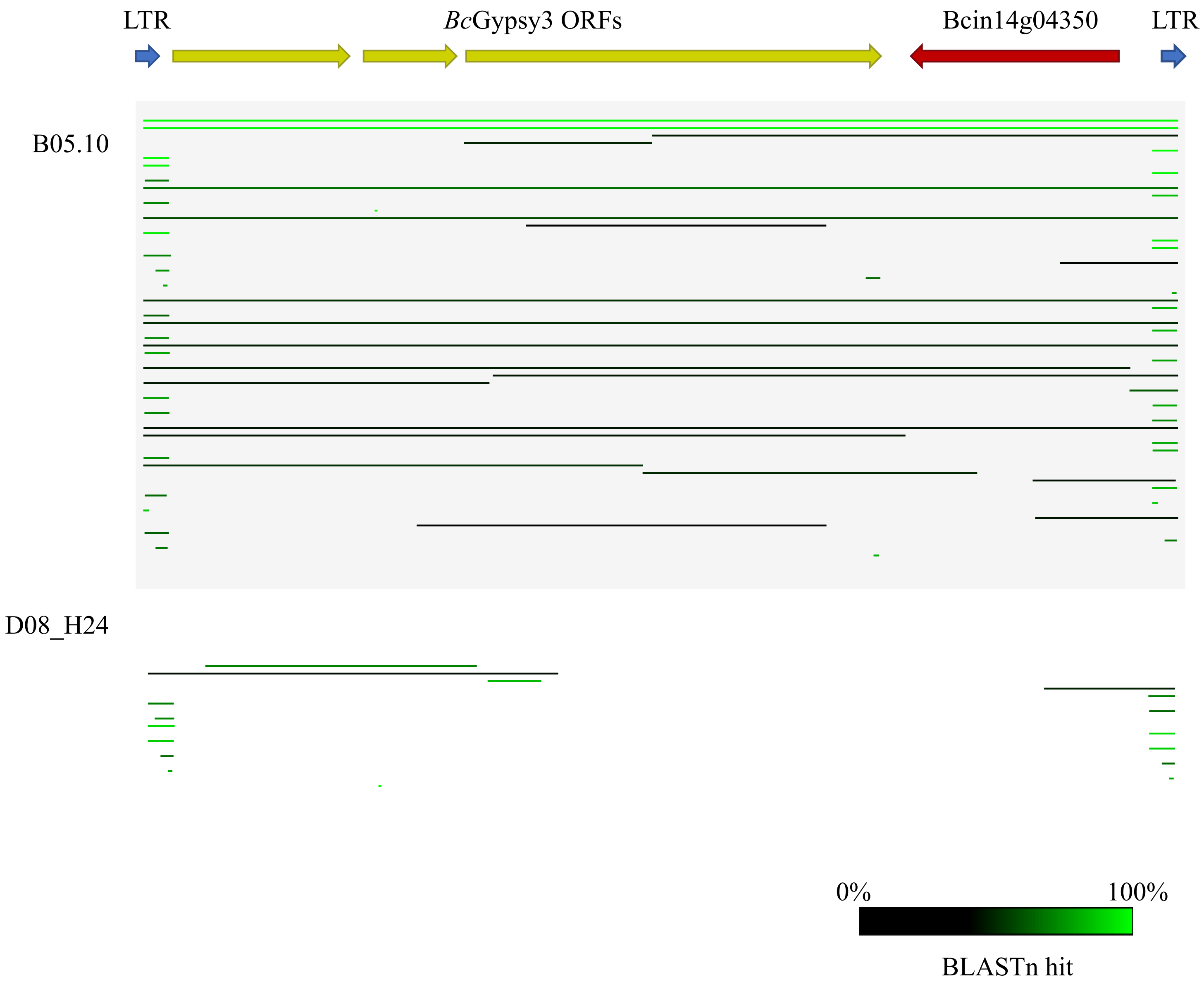

Figure S9: BLASTn search of *BcGypsy3* in B05.10 and D08.H24

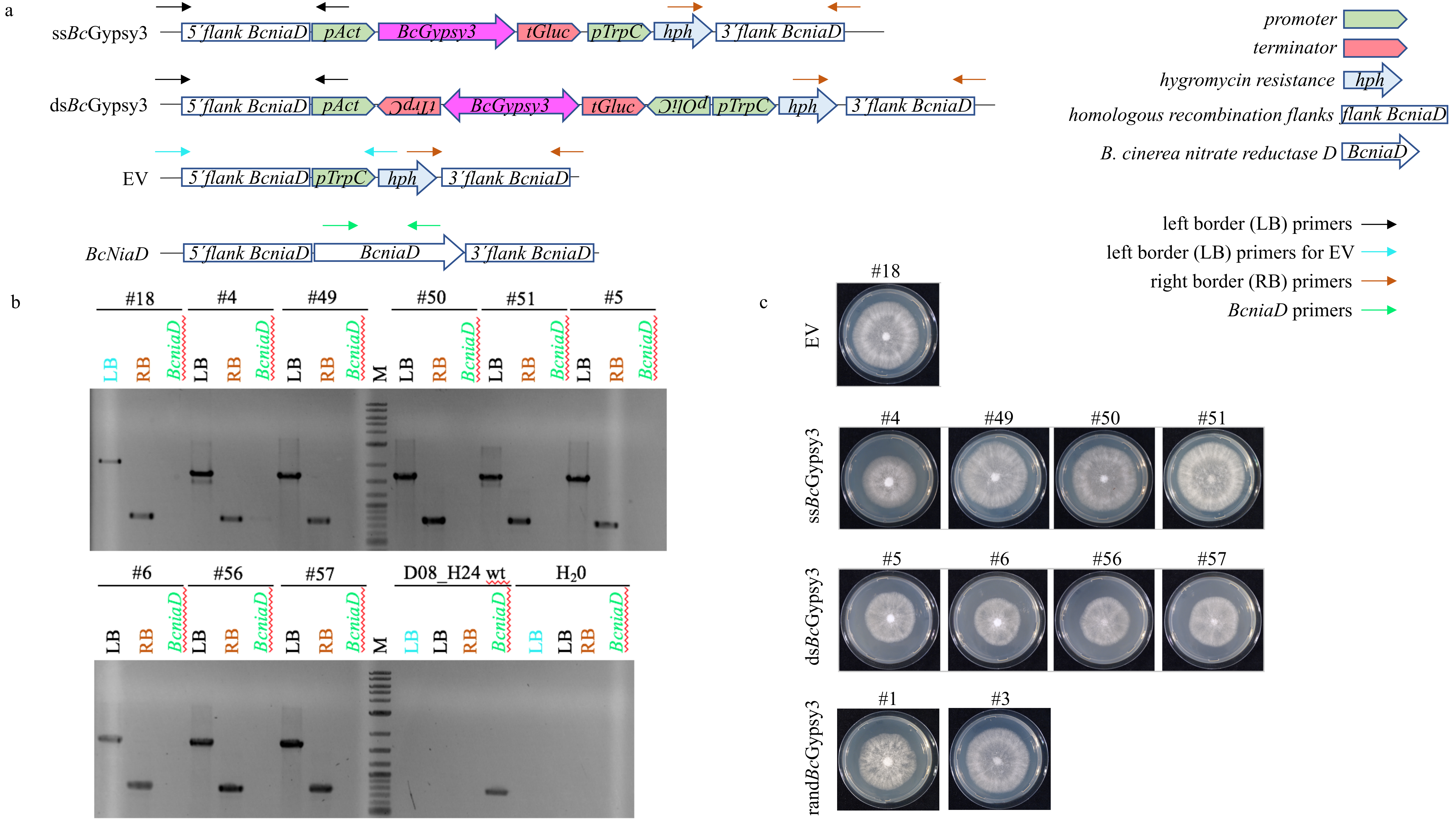

Figure S10: a) Cloning strategy and b) genotyping PCR of the integrated *BcGypsy3* transgene into the *BcniaD* locus. M: 1 kb DNA ladder marker. c) Morphological phenotypes of D08_H24 *Bc*Gypsy3 transformants.

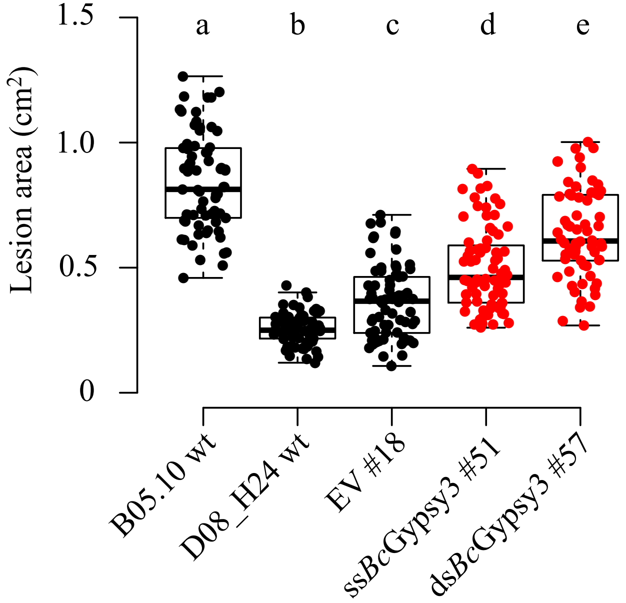

Figure S11: Replication of tomato leaf pathogenicity assay with dropped spore suspension of *Bc*Gypsy3 transformants and *B. cinerea* D08_H24 and B05.10 wt strains. Lesion area of > 20 infection sites were quantified at 48 hpi. Significant difference is indicated by letters, and was tested by one-way ANOVA using Tukey HSD test with $p < 0.05$.

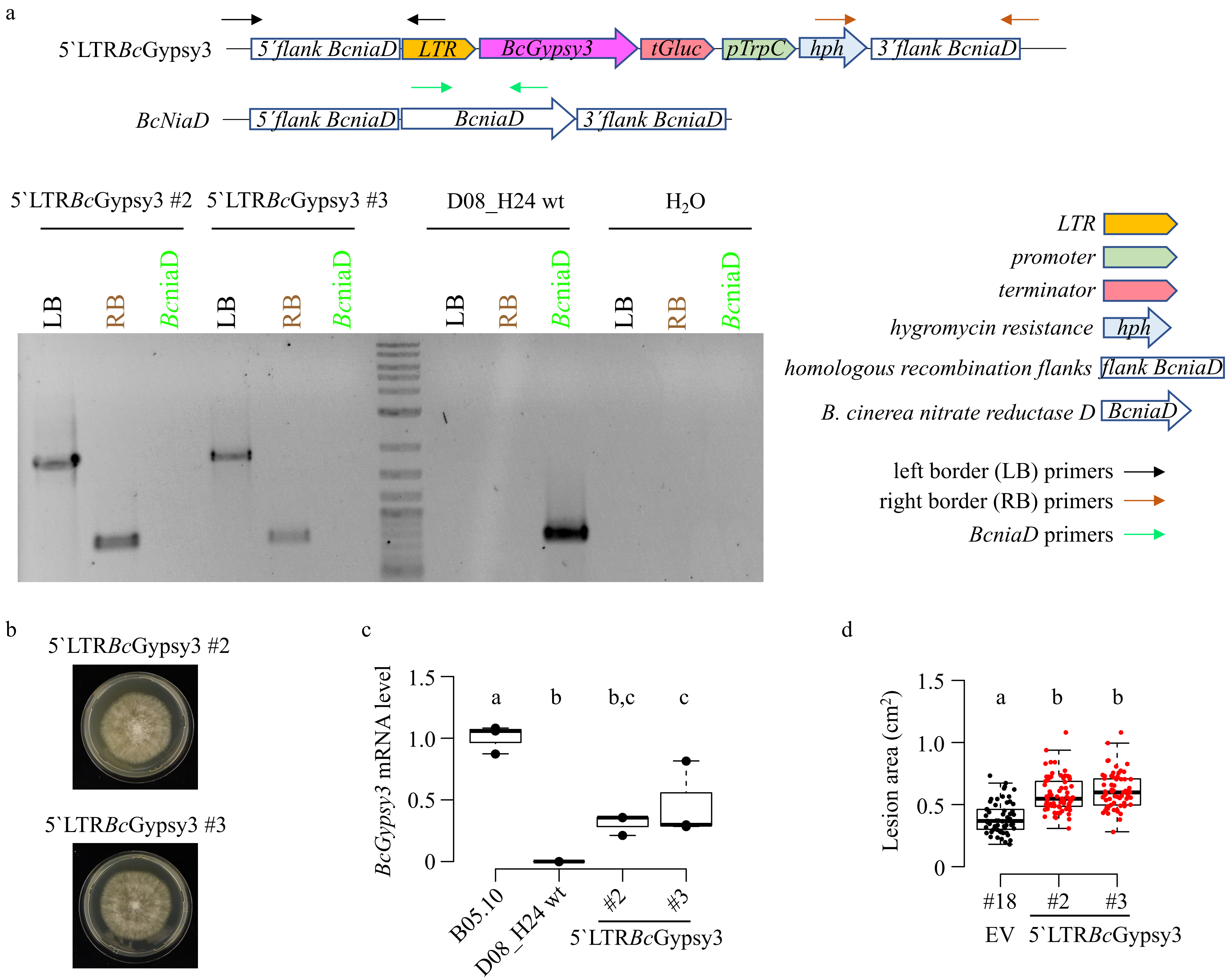

Figure S12: a) Cloning strategy and genotyping PCR of the integrated 5`LTR*BcGypsy3* transgene into the *BcniaD* locus. M: 1 kb DNA ladder marker. b) Morphological phenotypes of D08_H24 5`LTR*Bc*Gypsy3 transformants. c) *BcGypsy3* RNA expression in 5`LTR*Bc*Gypsy3 (#2, #3). Data points represent three biological replicates. Significant difference as indicated by letters was tested by one-way ANOVA using Tukey HSD test with $p < 0.05$. d) Pathogenicity assay with dropped spore suspension of 5`LTR*Bc*Gypsy3 transformants or EV transformant (#18) on tomato leaves quantifying lesion area of > 50 infection sites at 48 hpi. Significant difference as indicated by letters was tested by one-way ANOVA using Tukey HSD test with $p < 0.01$.

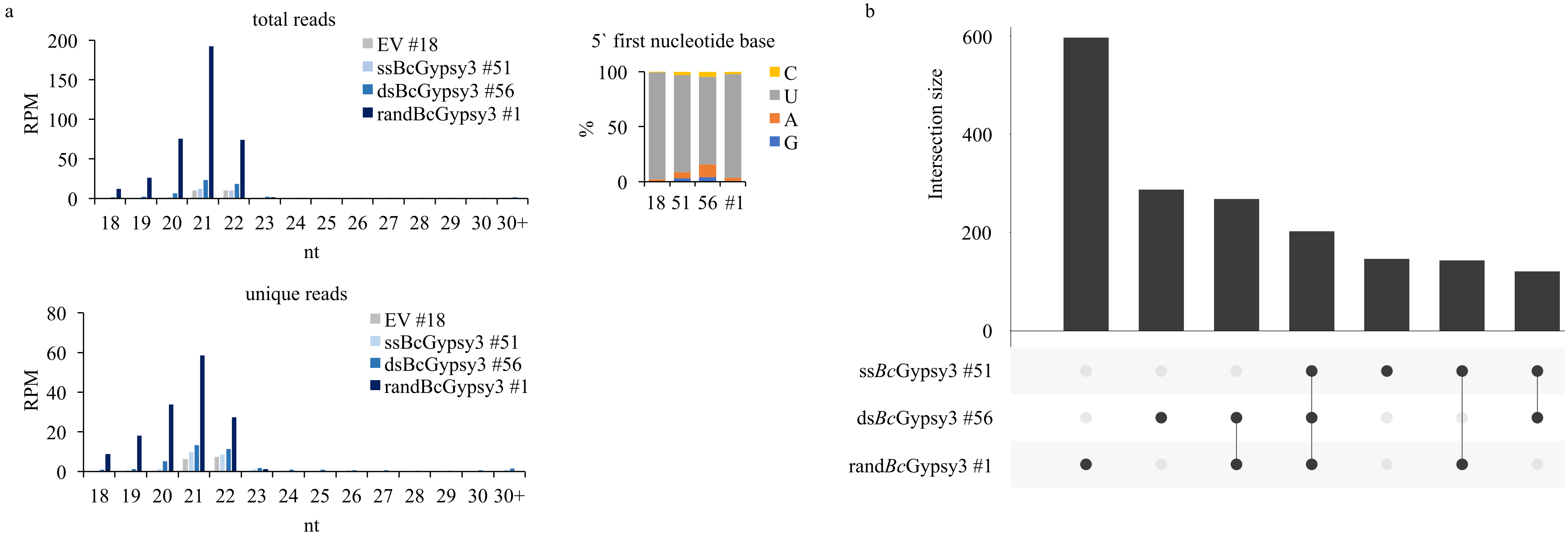

Figure S13: a) *Bc*sRNA size profiles and 5`end first nucleotide distribution of *Bc*Gypsy3 transformants. b) UpSet plot displaying the intersection size of *Bc*Gypsy3 *Bc*sRNAs among ss*Bc*Gypsy3 #51, ds*Bc*Gypsy3 #56 and rand*Bc*Gypsy #1 strains.

a

```
BcsRNA3.2 3'GUGGAUGUUCUAGGUGUUACA 5'
            ||::|| ||||||||| ||||
SlMPKKK4  5'CAUUUAAAAGAUCCACCAUGU 3'

BcsRNA3.1 3'CGGGUGGAUGUUCUAGGUGUU 5'
             |||||||:||| ||||||:|
Slhse1    5'ACCCACCUGCAACAUCCACGA 3'

BcsRNA20 3'UUAGUCUUUUUGUUCUUGUGAU 5'
           |:| |||||||||| ||:||||
SlBhlh63 5'AGUAAGAAAAACAAUAAUACUA 3'
```

b

```
BcsRNA3.2 3'UGGAUGUUCUAGGUGUUACAU 5'
            |:| | |||||:||||||||
AtMPK1    5'AUCAAGAAGAUUCACAAUGUU 3'

BcsRNA3.2 3'UGGAUGUUCUAGGUGUUACAU 5'
            |:| | ||||||||||||||:
AtMPK2    5'AUCAAGAAGAUCCACAAUGUG 3'

BcsRNA3.1 3'CGGGUGGAUGUUCUAGGUGUU 5'
            |||:| |||||||| |||||
AtPRXIIF  5'GCCUAGCUACAAGAGCCACAU 3'
```

c

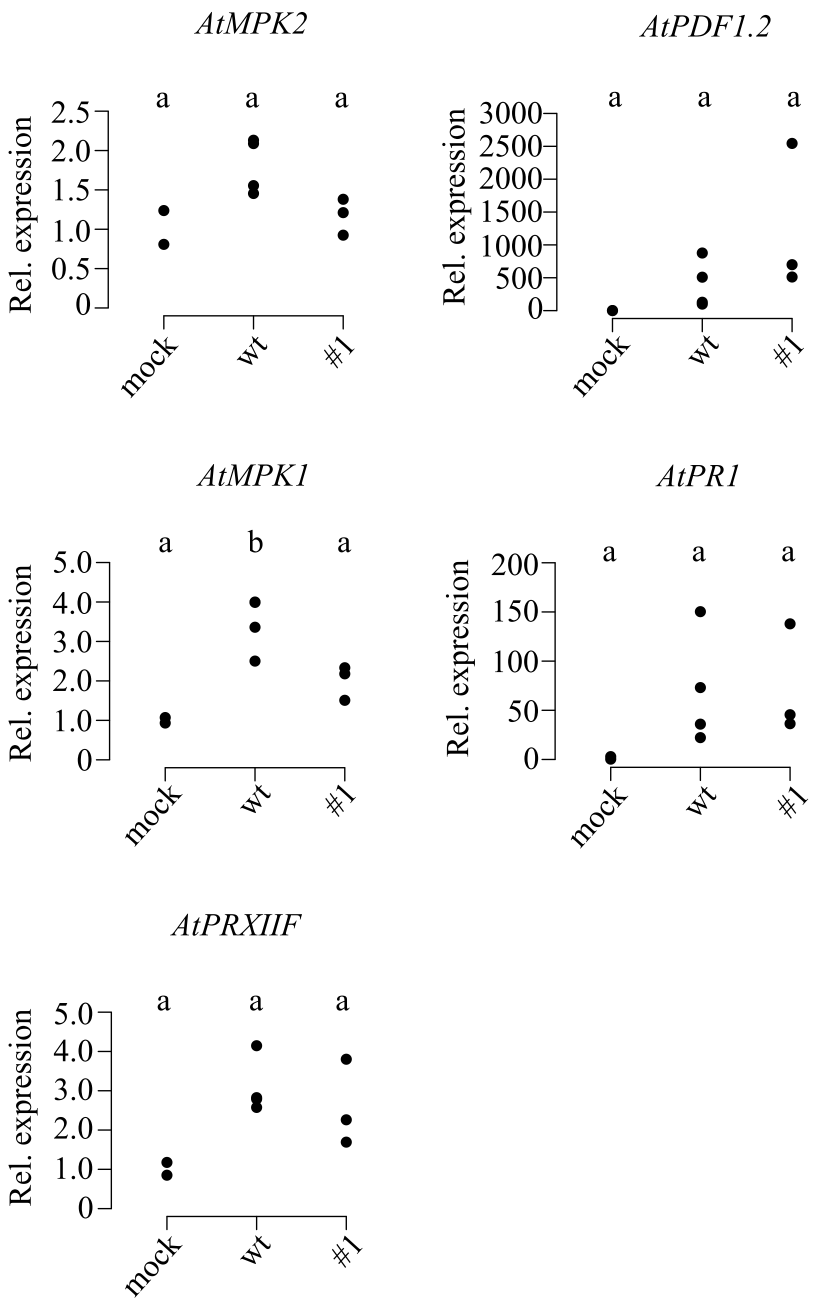

Figure S14: a-b) Sequence alignment of *Bc*sRNA effectors to *S. lycopersicum* (*Sl*) or *A. thaliana* (*At*) mRNA target candidates. c) qRT-PCR analysis of *A. thaliana* target mRNAs after no-infection (mock) or after infection with D08_H24 wt or rand*Bc*Gypsy3 #1. *AtPDF1.2* and *AtPR1* were used as *B. cinerea*-inducible genes in *A. thaliana* and these gene were not predicted targets of *Bc*sRNAs. Each data point represents a biological replicate.

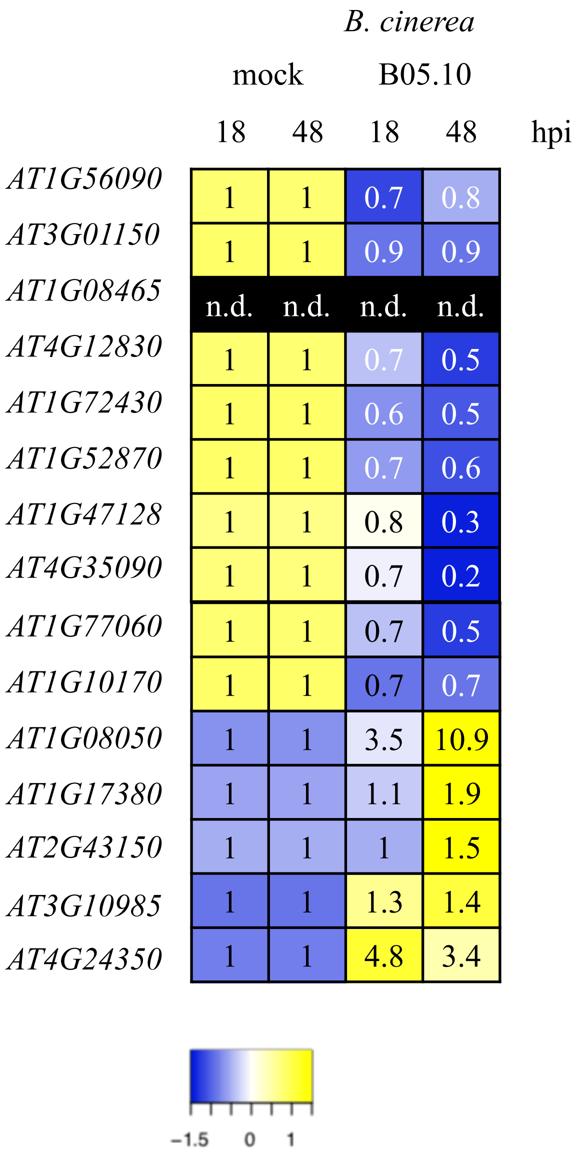

Figure S15: Change-fold factors of expressed genes found in *A. thaliana* mock versus *B. cinerea* infection, according to the BAR database.
