# Supplementary material for: Retrotransposons as pathogenicity factors of the plant pathogenic fungus Botrytis cinerea
Source: Genome Biol. 2021 Aug 16;22:225. doi: 10.1186/s13059-021-02446-4 (PMC8365987; doi:10.1186/s13059-021-02446-4)
Supplement: Supplementary file 3 — Additional file 3:. Review history. [file 13059_2021_2446_MOESM3_ESM.docx]

Review History

**First round of review**

**Reviewer 1**

**Are you able to assess all statistics in the manuscript, including the appropriateness of statistical tests used?** Yes, and I have assessed the statistics in my report.

**Comments to author:**

This manuscript analyzes multiple strains of the plant pathogenic fungus Botrytis cinerea. The six strains analyzed vary in their virulence on tomato plant hosts. The manuscript shows that high virulence correlates with high accumulation of fungal small RNAs from certain retrotransposon families. Less virulent Botrytis strains have much less of these retrotransposon-deived small RNAs. Both PCR-based and whole-genome-based methods show that the copy number of the sRNA-producing LTR-retrotransposons varies between the strains; less virulent strains have much fewer of the sRNA-producing retrotransposons. This is of particular interest because previous work has shown that some of these Botrytis sRNAs that come from retrotransposons can enter plants and target plant mRNAs involved in defense responses. The final experiments make a direct test of the transposon / virulence connection by transforming a strong sRNA-producing transposon from a highly aggressive strain into a weaker strain that naturally lacks the transposon. These transformants indeed show increased virulence that again correlated with transposon-derived sRNA abundance. The strength of this effect varied based on the details of the transgene promoter and/or the transgene integration site, but the effect was clear.

This is an interesting and important study. The connection between transposons and Botrytis virulence is clear, both from the correlations and from the direct transgenic experiment. It will be of wide interest in the field. The conclusions are sound and well supported by the data. I have some minor comments for the authors to consider:

1. It wasn't clear to me why the transgenic experiment exlcuded the LTRs of the element. Wouldn't that be the more natural expeirment? (introduction of the fully functional element into the less aggressive strain). If this experiment is feasible, it would be great information to have. However I do not feel this needs to be a firm requirement for publication; the exisiting data do support the conclusions.

2. Data availability: The manuscript includes small RNA-seq, mRNA-seq and whole genome assemblies. The manuscript says that these data "will be submitted" to SRA, but this is not adequate for peer review. All data must be archived and the SRA accession(s) given in the manuscript prior to acceptance. The authors can request a reviewer-only access token to allow peer review while keeping the data private until actual publication. This should be a firm condition for manuscript acceptance.

3. Minor things:

Abstract : "Retrotransposons are genetic elements inducing mutations in all
domains of life." -- maybe back off on this a bit. What about viruses? Are you sure about weird organisms like Archaea? Universal statements like this are chancy.

Abstract : "...retrotransposons become temporarily
active during epigenetic reprogramming and cellular stress response,..." Again, maybe back off on this a little. Better to say "...retrotransposons *can* become temporarily ... ", so that the implication is not that EVERY retrotransposon does this.

p. 3 line 59 : Figure 1B is cited before Figure 1A is mentioned. Shouldn't they be in sequence, or the panel numbers of the figure switched?

p. 5 line 22 : section heading .. "retrotransposons" should instead be singular "retrotransposon".

**Reviewer 2**

**Are you able to assess all statistics in the manuscript, including the appropriateness of statistical tests used?** Yes, and I have assessed the statistics in my report.

**Comments to author:**

In the article "Retrotransposons as pathogenicity factors of the plant pathogenic fungus Botrytis cinerea", authors show evidences that retrotransposons are used as pathogenicity factors by the plant pathogen B. cinerea, and identified several sRNAs as the effectors of this pathogenicity. Here are my comments that need to be addressed before acceptance:

1.     Do all Bc strain have other types of retrotransposons that are not included in the 6 supefamilies analyzed by PCR? It is strange to me that all the less aggressive strains do not contain any of the studies LTRs. What phenomenon would explain the complete loss of all these TE?

2.     How accurate is the amplification with the used primers? Would it be better to try partially degenerated primers?

3.     Authors describe in the methods "Sequence raw data are deposited at the NCBI SRA server (BioProject accession: tba)" and "Processed raw data are deposited at the NCBI SRA server (BioProject accession: tba)"  please provide the number and token for revision before publication.

4.     Table S4: Only a 22% of the reads generated from D08_H24 mapped to the Bc genome. Could this be an explanation of why no TEs were found?

5.     Figure 2: The authors make a circular representation of sRNA origin in panel B. The human eye is not very good a differentiating circular proportion. Please, include the relative and/or absolute percentage in each graph.

6.     Figure 2: The authors present a sRNA size distribution on panel C. However, it is not very clear to me what RPM x1000 means. Please, present the graphs in RPM and include the standard sizes, including 18nt and a 30+ category.

7.     Figure 2: Please, include a similar size distribution graph, for total sRNAs and LTR-derived sRNAs, including only distinct sequences. This will give the reader an idea of the complexity of the reads. Additionally, are the sRNA LTR-derived sequences found in the different aggressive lines common? Maybe an additional UpSet plot would be good.

8.     Figure 3B. There is no observable difference in the genome size of the three strains. However, there is a big difference in the size of the LTRs. Does this difference exist in other TE families? Is the loss of LTRs compensated with the expansion of other repetitive elements?

9.     In figure S10, the light blue arrows are missing from the legend.

10.     Figure 4B: Please, include the positive and negative controls in the lesion area graphs, WT B05 and D08.

11.     Figure S11: Please, include a similar size distribution graph, for total sRNAs and LTR-derived sRNAs, including only distinct sequences. As well as an upset plot of common sequences.

12.     Figure $#: Please, use a box plot format for this graph, similar to 4A, for a better statistical understanding of the significance.

13.     In "quantify gene expression of the known BcsRNA3.1 and BcsRNA3.2 target genes AtMPK1, AtMPK2 and a Cell wall-associated kinase (AtWAK) (Fig.S11B)" I think authors mean Fig S12B. Please, correct this here and in the next citations.

14.     Figure 4F: Please, change the colors used in this figure, since some color deficiencies will not be able to see the difference between red and green. Please, test the new figure version in some kind of simulators (https://www.color-blindness.com/coblis-color-blindness-simulator/) . Also, please add the gene name or code to the heat map. It remains unclear if the direct targes of the BcsRNAs identified here appear down-regulated in the RNAseq experiments.

**Authors Response**

**Point-by-point response to the reviewers` comments**

**Comments of Reviewer 1:**

**Reviewer 1:** 1. It wasn't clear to me why the transgenic experiment excluded the LTRs of the element. Wouldn't that be the more natural experiment? (introduction of the fully functional element into the less aggressive strain). If this experiment is feasible, it would be great information to have. However, I do not feel this needs to be a firm requirement for publication; the existing data do support the conclusions.

**Authors:** We thank the reviewer for this comment. We decided to exclude LTR elements from our *BcGypsy3* transgene constructs, because these typically contain binding sites of tRNA primers that initiate reverse transcription and transposon transposition (Murata et al., 2007), and because *Bc*sRNAs did not derive from the LTR regions, based on our small RNA-seq analysis (Fig.S2B). However, we agree with the reviewer that including LTRs in this experiment would reflect a more natural situation. We now provide additional data of transgenic D08_H24 strains carrying a 5`LTR element instead of an ectopic promoter. We have tested two independent transformants for the expression of *BcGypsy3* by qRT-PCR and in pathogen assays on tomato leaves. The results are now displayed in the new Figure S12. In brief, these two additional transgenic strains exhibited clear mRNA expression of Bc*Gypsy3* and significantly increased aggressiveness compared to EV control #18 and D08_H24 WT. This result further confirmed that *Bc*Gypsy3 contributed to pathogenicity of *B. cinerea*.

**Reviewer 1:** 2. Data availability: The manuscript includes small RNA-seq, mRNA-seq and whole genome assemblies. The manuscript says that these data "will be submitted" to SRA, but this is not adequate for peer review. All data must be archived and the SRA accession(s) given in the manuscript prior to acceptance. The authors can request a reviewer-only access token to allow peer review while keeping the data private until actual publication. This should be a firm condition for manuscript acceptance.

**Authors:** We thank the reviewer for this comment. We have now uploaded all data to the public NCBI SRA and Genome servers in the BioProject PRJNA730711. We now give this reference ID in the manuscript text.

Minor things:

**Reviewer 1**: Abstract: "Retrotransposons are genetic elements inducing mutations in all
domains of life." -- maybe back off on this a bit. What about viruses? Are you sure about weird organisms like Archaea? Universal statements like this are chancy.

**Authors:** We thank the reviewer for his thoughtful comment. We have cross-checked the given literature and found a report about retroelements existing in *Archaeabacteria* (Dai and Zimmerly, 2003) and now include this citation in our manuscript. Since retrotransposons originate from retrovirus, they could be considered as the same. To make our statement clearer, we have rephrased the sentence into: "Retrotransposons are genetic elements inducing mutations in the bacteria, archaea and eukaryote domains of life."

**Reviewer 1:** Abstract: "...retrotransposons become temporarily active during epigenetic reprogramming and cellular stress response,..." Again, maybe back off on this a little. Better to say "...retrotransposons *can* become temporarily ... ", so that the implication is not that EVERY retrotransposon does this.

**Authors:** We thank the reviewer for this comment. We have changed the statement following the reviewer`s suggestion.

**Reviewer 1:** p. 3 line 59: Figure 1B is cited before Figure 1A is mentioned. Shouldn't they be in sequence, or the panel numbers of the figure switched?

**Authors:** We thank the reviewer for this comment. We now cite Figure 1A the first time in line 60 and Figure 1B in line 67, thus the order should be correct.

**Reviewer 1:** p. 5 line 22: section heading... "retrotransposons" should instead be singular "retrotransposon".

**Authors:** We thank the reviewer for spotting this mistake. We have now corrected the heading, accordingly.

**Comments of Reviewer 2:**

**Reviewer 2:** 1. Do all Bc strain have other types of retrotransposons that are not included in the 6 supefamilies analyzed by PCR? It is strange to me that all the less aggressive strains do not contain any of the studies LTRs. What phenomenon would explain the complete loss of all these TE?

**Authors:** We thank the reviewer for this thoughtful comment. The PCR results shown in the Figure 1D based on primers that were derived from LTR retrotransposon consensus sequences for each of the six subfamilies, as identified in the strain B05.10 by REPET analysis. Our explanation why these primers did not produce any PCR product for the strains D08_H24 and D14_KF is that these strains comprise only RIPed AT-rich retrotransposons, as we revealed this by our WGS approach. Indeed, BLAST search of these primer sequences within the genome sequences of D08_H24 and D14_KF did not result in any match. Moreover, REPET and BLASTn analyses of the D08_H24 and D14_KF genome sequences did not reveal any other type of LTR retrotransposon besides the described AT-rich copies of the beforehand introduced *Bc*Gypsy and *Bc*Copia subfamilies. Since the strain D13_TS was also negative for all tested PCR primers, we presume that this strain also contains only RIPed AT-rich LTR retrotransposons.

**Reviewer 2:** 2. How accurate is the amplification with the used primers? Would it be better to try partially degenerated primers?

**Authors:** We thank the reviewer for this suggestion. We now have tested degenerated primers that include possible C/T and G/A wobbles, reflecting the RIP mutations found at these sequences. The PCR did not result in any detectable PCR products (see Figure R1 at the end of the response letter), possibly due to very high AT content of the degenerated primers.

**Reviewer 2:** 3. Authors describe in the methods "Sequence raw data are deposited at the NCBI SRA server (BioProject accession: tba)" and "Processed raw data are deposited at the NCBI SRA server (BioProject accession: tba)" please provide the number and token for revision before publication.

**Authors:** We thank the reviewer for this comment and apologize for the missing links. We now have uploaded all data to the public NCBI SRA and Genome servers under the BioProject PRJNA730711. We now give this reference within the manuscript text.

**Reviewer 2:** 4. Table S4: Only a 22% of the reads generated from D08_H24 mapped to the Bc genome. Could this be an explanation of why no TEs were found?

**Authors:**

We are convinced that this cannot be an explanation why no TEs were found, because this would mean that our D08_H24 genome assembly would be insufficient. However, our assembly statistics prove a high-quality assembly: we calculated an 800x coverage referring to the B05.10 reference genome (Van Kan et al., 2017) by Nanopore sequencing. Our assembly of the D08_H24 genome comprised an N50 = 2.6 Mb indicating high assembly quality for transposon annotation. Based on this assembly, we annotated retrotransposons in D08_H24 using REPET and BLASTn tools, as presented in Figure 3b and in Table S5. In addition, we re-run mapping of the D08_H24 small RNA reads to the D08_H24 genome using the BWA mapper (Li and Durbin, 2009) with 1, 2, and 3 mismatches. Indeed, mismatch of 3 led to 79.8% coverage number (Table R1); nevertheless, the number of retrotransposon-derived small RNAs in D08_H24 stayed significantly low, compared to the aggressive strains B05.10, N11_KW and D13_TF.

**Reviewer 2:** Figure 2: The authors make a circular representation of sRNA origin in panel B. The human eye is not very good a differentiating circular proportion. Please, include the relative and/or absolute percentage in each graph.

**Authors:** We thank the reviewer for this suggestion. We now provide the relative percentage in Figure 2B.

**Reviewer 2:** Figure 2: The authors present a sRNA size distribution on panel C. However, it is not very clear to me what RPM x1000 means. Please, present the graphs in RPM and include the standard sizes, including 18nt and a 30+ category.

**Authors:** We agree with the reviewer and have now changed the graphs to give RPM units as well as to include the 18 and 30+ size categories in the Figure 2C and D.

**Reviewer 2:** Figure 2: Please, include a similar size distribution graph, for total sRNAs and LTR-derived sRNAs, including only distinct sequences. This will give the reader an idea of the complexity of the reads.

**Authors:** We thank the reviewer for this suggestion. We now present two new graphs displaying the size distribution of unique reads for total sRNAs as well as LTR retrotransposon-derived sRNAs in the new Figure S4.

**Reviewer 2:** Additionally, are the sRNA LTR-derived sequences found in the different aggressive lines common? Maybe an additional UpSet plot would be good.

**Authors:** We agree with the reviewer. We now include an UpSet plot presented, as suggested by the reviewer, in the new Figure S4.

**Reviewer 2:** Figure 3B. There is no observable difference in the genome size of the three strains. However, there is a big difference in the size of the LTRs. Does this difference exist in other TE families? Is the loss of LTRs compensated with the expansion of other repetitive elements?

**Authors:** We thank the reviewer for spotting this point. The results regarding the detection of other transposon types by the REPET analysis for the three strains B05.10, D08_H24 and D14_KF are presented in the Table R2 at the bottom of this response letter. We found that accumulation of DNA transposons and other types of repetitive elements that were not further classified (noCat) was also reduced in D08_H24 and D14_KF compared to B05.10, similar to LTR retrotransposons. Since the reduced accumulation of transposable elements is not reflected in the genome sizes, as given in Figure 2B, we would presume that other types of DNA have compensated for the reduction of transposons.

**Reviewer 2:** In figure S10, the light blue arrows are missing from the legend.

**Authors:** We thank the reviewer for spotting this mistake. We have fixed the legend, accordingly.

**Reviewer 2:** Figure 4B: Please, include the positive and negative controls in the lesion area graphs, WT B05 and D08.

**Authors:** We agree with the reviewer`s suggestion. We have now repeated the infection assay including the WT B05 and D08 and the lesion area data are now presented in the new Figure S11.

**Reviewer 2:** Figure S11: Please, include a similar size distribution graph, for total sRNAs and LTR-derived sRNAs, including only distinct sequences. As well as an upset plot of common sequences.

**Authors:** We thank the reviewer for this comment. We now provide an updated size profile of total and distinct sequences, as well as an UpSet plot, as suggested by the reviewer, in the Figure S13 (formerly Figure S11).

**Reviewer 2:** Figure $#: Please, use a box plot format for this graph, similar to 4A, for a better statistical understanding of the significance.

**Authors:** We have changed Figure 4E into box plots, as suggested by the reviewer.

**Reviewer 2:** In "quantify gene expression of the known BcsRNA3.1 and BcsRNA3.2 target genes AtMPK1, AtMPK2 and a Cell wall-associated kinase (AtWAK) (Fig.S11B)" I think authors mean Fig S12B. Please, correct this here and in the next citations.

**Authors:** We thank the reviewer for spotting this mistake. We have now corrected the Figure reference.

**Reviewer 2:** Figure 4F: Please, change the colors used in this figure, since some color deficiencies will not be able to see the difference between red and green. Please, test the new figure version in some kind of simulators (<https://www.color-blindness.com/coblis-color-blindness-simulator/>). Also, please add the gene name or code to the heat map. It remains unclear if the direct targes of the BcsRNAs identified here appear down-regulated in the RNAseq experiments.

**Authors:** We thank the reviewer for this important comment. We have changed the colours of the heat map in Figure 4F, according the colour blindness test.

**References:**

**Dai, L., and Zimmerly, S.** (2003). ORF-less and reverse-transcriptase-encoding group II introns in archaebacteria, with a pattern of homing into related group II intron ORFs. RNA **9,** 14-19.

**Li, H., and Durbin, R.** (2009). Fast and accurate short read alignment with Burrows-Wheeler transform. Bioinformatics **25,** 1754-1760.

**Murata, T., Kadotani, N., Yamaguchi, M., Tosa, Y., Mayama, S., and Nakayashiki, H.** (2007). siRNA-dependent and -independent post-transcriptional cosuppression of the LTR-retrotransposon MAGGY in the phytopathogenic fungus Magnaporthe oryzae. Nucleic Acids Res **35,** 5987-5994.

**Van Kan, J.A., Stassen, J.H., Mosbach, A., Van Der Lee, T.A., Faino, L., Farmer, A.D., Papasotiriou, D.G., Zhou, S., Seidl, M.F., Cottam, E., Edel, D., Hahn, M., Schwartz, D.C., Dietrich, R.A., Widdison, S., and Scalliet, G.** (2017). A gapless genome sequence of the fungus *Botrytis cinerea*. Mol Plant Pathol **18,** 75-89.

**Figure R1:** *Bc*Gypsy1 degenerated primers PCR. Non-generated primers were used as a positive control. Similarly, degenerated PCR primers for BcGypsy2-BcGypsy4 and BcCopia1 and BcCopia2 did not show any PCR products.

**Table R1:** Coverage (in %) of small RNA reads to the reference genome and LTR retrotransposons of D08_H24 using the BWA aligner.

| mismatches | genome | LTR retrotransposon |
| --- | --- | --- |
| 1 | 29.2 | 0.005 |
| 2 | 38.6 | 0.02 |
| 3 | 79.8 | 0.27 |

**Table R2:** Accumulation (in kb) of class 2 transposable elements in the three genome sequence strains:

|  | B05.10 | D08_H24 | D14_KF |
| --- | --- | --- | --- |
| Class 2 DNA transposons | 283 kb | 23 kb | 226 kb |
| noCat | 306 kb | - | 6 kb |

**Second round of review**

**Reviewer 1**

This revision addressed my comments very well. This is a very interesting study and I look forward to seeing it published soon.

**Reviewer 2**

I thank the authors fro reviewing the manuscript and figure and addressing our concerns. I think the manuscript has been improved and is ready for publication. However, even if the authors responses about Figure S13 and Figure 3B are valid, they have not been introduced in the main text. Please, add the discussed points regarding these figures in the text.
